# Anxiety and Depression Symptom Level and Psychotherapy Use Among Music and Art Students Compared to the General Student Population

**DOI:** 10.3389/fpsyg.2021.607927

**Published:** 2021-06-28

**Authors:** Jonas Vaag, Ottar Bjerkeset, Børge Sivertsen

**Affiliations:** ^1^Faculty of Nursing and Health Sciences, Nord University, Bodø, Norway; ^2^Department of Psychology, Faculty of Social and Educational Sciences, Norwegian University of Science and Technology, Trondheim, Norway; ^3^Department of Mental Health, Faculty of Medicine and Health Sciences, Norwegian University of Science and Technology, Trondheim, Norway; ^4^Department of Health Promotion, Norwegian Institute of Public Health, Bergen, Norway; ^5^Department of Research and Innovation, Fonna Hospital Trust, Haugesund, Norway

**Keywords:** performing arts, music students, anxiety, depression, performing arts medicine

## Abstract

**Purpose:** Previous epidemiological studies have shown higher levels of anxiety and depressive symptoms among professional musicians, compared to the general workforce. Similar findings have been observed for psychotherapy use among musicians. To date, large-scale investigations of prevalence rates among music and arts students are lacking.

**Methods:** Eight hundred and eighty students from music and arts institutions and faculties were derived from a national health student survey for higher education in Norway (the SHoT study). They were compared to a sample of the general student population (*n* = 48,729). We used logistic regression analysis, adjusting for age, sex, and semesters of study.

**Results:** Music and arts students reported higher rates of anxiety [OR 1.60 (1.38–1.85), Prevalence difference (PD) 9.6 (6.3–12.8)] and depression symptoms [OR 1.41 (1.22–1.62), PD 7.9 (4.5–11.2)] compared to the general student force. Similar patterns were observed for self-reported mental disorders [OR 1.71 (1.46–2.01), PD 8.1 (5.3–11.0)], as well as psychotherapy use [OR 1.91 (1.60–2.29), PD 7.4 (4.9–9.9)] in music and arts students.

**Conclusions:** Our findings are consistent with studies comparing musicians to the general workforce, and indicate that challenges also exist at student level, and not only after becoming a professional in the performing arts, which is important when planning health-related measures. These findings have the potential to inform on health promotion and services in the educational system.

## Introduction

The creative industries have been described by a high degree of occupational stress (Wills and Cooper, 1987; Middlestadt and Fishbein, 1988; Smith et al., 2000; Iñesta et al., 2008; Gross and Musgrave, 2020), and recent studies have suggested that the psychosocial work environment of musicians are more demanding than in most other occupations (Holst et al., 2012; Burak and Atabek, 2019; Detari et al., 2020; Gross and Musgrave, 2020). In addition to the industry being described as potentially harmful, there are also studies indicating vulnerability at the individual level. In example, creativity, a prerequisite for many forms of artistic and musical performances, has been shown to be associated with increased risk of affective disorders (Akiskal et al., 2005; Mula and Trimble, 2009; Kyaga et al., 2013). Compared to the general workforce, recent studies have shown that professional musicians suffer from more symptoms of anxiety and depression (Vaag et al., 2016a,b; Gross and Musgrave, 2020; Kegelaers et al., 2021), sleep problems (Vaag et al., 2015), and use more psychotherapy (Vaag et al., 2016b) as well as manual, complementary and alternative healthcare services (Vaag and Bjerkeset, 2017). As far back as the nineteen-twenties, Rogers (1926) asked the paradoxical question that, even though engagement in music seems to be health promotive, having it as a profession could have negative effects. This paradox is also the theme of Gross and Musgrave's (2020) book “Can Music Make You Sick?,” where they, based on large-scale investigations in the UK, argue for a transformation of the industry. But what about the educational system which prepare students for a career within arts and music?

The existing literature implies there is an increased level of symptoms of anxiety and depression among music students, compared to the general student population. In a study by Spahn et al. (2004), 247 music students were compared to 266 medical students, 71 psychology students, and 71 sports students. The authors found higher levels of anxiety and depression, compared to the other students. In a study by Ginsborg et al. (2009), investigating health-promoting behaviors and general health among music (*n* = 198) and non-music (*n* = 65) students in the UK, music students reported lower degrees of self-efficacy and self-regulation. Further, a study from South Africa by Panebianco-Warrens et al. (2015) also found similar results for psychosocial well-being of undergraduate music students (*n* = 144).

In conjunction with the growing amount of research indicating elevated levels of ill health among professional musicians and music students, there has been an increased interest in health promotion and preventive measures within music and art education (Araújo et al., 2017; Perkins et al., 2017; Matei et al., 2018; Aalberg et al., 2019). Kegelaers et al. (2020) recently published a small study (*n* = 64) suggesting that anxiety and depression symptoms are not only highly prevalent among music students, their symptom load is even higher than observed among professional musicians. In contrast, an uncontrolled US study by Wristen (2013) did not find higher levels anxiety and depression among 287 music students compared to estimates presented in other studies. The knowledge about health risk and illness in the student population has, to this date, yet to be investigated using large-scale samples comparing music and arts students to the general student population.

By using data from a national student health survey for higher education in Norway, the aim of the current study was to investigate the prevalence of symptoms and self-reported disorders of anxiety and depression among 880 music and art students, compared to the general student population.

## Methods

### Participants and Setting

Data stem from the SHoT2018 study (Students' Health and Well-being Study), a national student survey for higher education in Norway, initiated by the three largest student welfare organizations (Sammen [Bergen and surrounding area], Sit [Trondheim and surrounding area], and SiO [Oslo and Akershus]). Data for the SHoT2018 was collected electronically through a web-based platform. Details of the study has been published elsewhere (Sivertsen et al., 2019), but in short, the SHoT2018 was conducted between February 6 and April 5, 2018, and invited all fulltime Norwegian students aged 18–35 years pursuing higher education (both in Norway and abroad). In all, 162,512 students fulfilled the inclusion criteria, of whom 50,054 students completed the online questionnaires, yielding a response rate of 30.8%.

#### Ethics

The SHoT2018 study was approved by the Regional Committee for Medical and Health Research Ethics in Western Norway (no. 2017/1176). An electronic informed consent was obtained after the participants had received a detailed introduction to the study.

### Sample of Music and Art Students

In order to derive music and art students from the total sample, we had to rely on demographic information regarding student affiliation to music and arts institutions and faculties. By using this information, we were able to sample 880 students with affiliation to institutions such as the Norwegian Academy of Music, The Academy Barratt Due, Oslo National Academy of the Arts, and The Norwegian University College of Dance, as well as music and art faculties within institutions such as Ansgar University College, Østfold University College, University of Agder, University of Bergen, University of Stavanger, and The Arctic University of Norway. Since we had to rely on the students' affiliations to different faculties, our categorization should be interpreted with caution. Based on names of faculties, we divided into three groups. The “Music student group” (*n* = 327) consisted of students that were affiliated to institutions and faculties that were designated to music-related studies (involving music education, music teacher education, and music therapy). The “Performing arts” group (*n* = 256) consisted of students from institutions and faculties that provided education within dance, theater, musical theater, as well as music performance (where the faculties were more broadly named and not music only). The “Arts faculty” group (*n* = 297) were derived from an even broader group of students from institutions and faculties designated to education within the arts (also including performing arts and music).

## Measures

### Symptoms of Anxiety and Depression

Symptoms of anxiety and depression were measured using the 25-item version of The Hopkins Symptom Checklist 25 (Derogatis et al., 1974; Strand et al., 2003). The HSCL-25 is a self-administered and widely used instrument measuring symptoms of anxiety and depression. It is derived from the HSCL-90 and consists of 25 statements regarding anxiety (10 items) and depression (15 items). Each item (symptom) is measured on a Likert-scale from 1 to 4, with the following response options (1) “Not at all,” (2) “A little,” (3) Quite a bit, and (4) “Extremely.” Previous recommendations from the use of the instrument has been to use a mean score above 1.75 as a cut-off defining prevalence of moderate to severe anxiety and depression symptoms (Strand et al., 2003), but recent studies on student population samples has deemed it more reasonable to use a higher cut-off when using student samples (Knapstad et al., 2019). We chose to use a cut-off of above 2.0 as an indicator of prevalence of severe symptoms of anxiety and depression. In the current study, we used both the total HSCL-25 score, as well as the anxiety and depression subscales.

### Mental Disorders and Use of Psychotherapy

Self-reported mental disorders were assessed by a pre-defined list adapted to fit this age-cohort. The list was based on a similar operationalization used in previous large population-based studies [the HUNT study (Krokstad et al., 2013)] and included several subcategories for most conditions/disorders (not listed here). For mental disorders, the list comprised the following specific disorders/group of disorders: ADHD, anxiety disorder, autism/Asperger, bipolar disorder, depression, PTSD (posttraumatic stress disorder), schizophrenia, personality disorder, eating disorder, Tourette's syndrome, obsessive-compulsive disorder (OCD), and other. The list did not come with a description of the included disorders/conditions. In the current study, the two most prevalent disorders, anxiety and depression, were included. The rationale for including both the HSCL-25 as well as self-reported mental disorders was to provide an assessment of overall symptom load (from the HSCL-25) and an indication of the presence of a disorder. Current use of psychiatrist and/or psychologist was also assessed by self-report and used as an indicator of psychotherapy use in our study.

### Demographic Covariates

We included sex, age, and semesters of study as demographic covariates in our study. This in order to control for the possibility of differences in distribution of these variables within the different institutions and faculties.

### Statistics

Data were analyzed using STATA, version 16.0 (StataCorp., 2011). We used descriptive statistics to quantify the symptom level of anxiety and depression, mental disorders and psychotherapy use. In addition, we conducted logistic regression analyses in order to assess the odds of having symptoms above cut-off level, mental disorders and psychotherapy use. We compared the sample of music and arts students, and their subgroups, to the general student sample. Analyses were done both crude and adjusted for age, sex, and semesters of study. In addition to calculating odds ratio (OR), we estimated prevalence differences (PD) with 95% confidence intervals (CI).

## Results

The sample of music and arts students were marginally younger than the rest of student population sample (mean age 23.0 and 23.3 years, respectively) (Table 1). There was no significant sex difference between the two samples.

**Table 1:** Prevalence estimates and distribution of demographic characteristics.

	**Student population**	**Music and arts students**	**ES^a^**
	**(*n* = 48,729)**	**(*n* = 880)**	
Age mean (CI)	23.3 (23.2–23.3)	23.0 (22.8–23.2)	0.08
**Age distribution (%)**			0.01
18–20	8,593 (17.9)	179 (20.6)	
21–22	15,050 (31.3)	288 (33.2)	
23–25	15,515 (32.3)	246 (28.3)	
26–28	5,562 (11.6)	105 (12.1)	
29–35	3,339 (7.0)	50 (5.8)	
Women (%)	33,526 (69.1)	607 (69.5)	
Semesters of study (CI)	6.2 (6.2–6.3)	5.8 (5.6–6.0)	0.13
**Symptoms of Anx/Dep (%)**	12,904 (26.5)	298 (34.0)	0.02
Anxiety	12,145 (25.0)	303 (34.6)	0.03
Depression	16,102 (33.2)	362 (41.3)	0.02
**Mental disorder (%)**	7,519 (15.4)	206 (23.4)	0.03
Anxiety disorder	4,868 (10.0)	140 (15.9)	0.03
Depressive disorder	5,375 (11.0)	135 (15.3)	0.02
Psychotherapy (%)	5,024 (10.3)	156 (17.7)	0.03

a *Effect size, in the form of Cramer's V on distribution, and Cohen's d on mean values. Effect size is listed if differences between groups are statistically significant (p < 0.05)*.

The proportion of students scoring above the cutoff for severe anxiety and depression symptoms was significantly higher among music and arts students (MA: 34.0%) compared to the general student population (SS): 26.5%). Similarly, the prevalence of mental disorders was also significantly higher among the music and arts students (23.4% vs. 15.4%, as well as psychotherapy use (MA: 17.7% / SS: 10.3%).

### Prevalence of Anxiety and Depression Symptoms

The adjusted prevalence difference of combined anxiety and depression symptoms was 7.2 percentage points (95% CI 4.0–10.4) higher among the music and arts students than in the general student population (Table 2). When comparing different subsamples of music and arts students, students from mixed arts and music faculties reported the highest prevalence difference in combined anxiety and depression symptoms [PD 11.0 (CI 5.3–16.6)]. The same tendencies were also seen for anxiety and depression symptoms separately.

**Table 2:** Logistic regression analysis of prevalence of symptoms of self-reported anxiety and depression symptoms in Norwegian music and arts students compared to the general student population.

		**Crude**		**Adjusted^a^**	
	**Prev. (%)**	**OR (CI)**	**PD (CI)**	**OR (CI)**	**PD (CI)**
**Symptoms of anx/dep**					
Student pop. (*n* = 48,621)	12,904 (26.5)	1 (Reference)		1 (Reference)	
All music and arts (*n* = 877)	298 (34.0)	1.42 (1.24 to 1.64)	7.4 (4.3 to 10.6)	1.42 (1.22 to 1.64)	7.2 (4.0 to 10.4)
Music (*n* = 327)	94 (28.8)	1.12 (0.88 to 1.42)	2.2 (−2.7 to 7.1)	1.24 (0.96 to 1.58)	4.2 (−0.9 to 9.4)
Perf. arts (*n* = 255)	90 (35.3)	1.51 (1.17 to 1.95)	8.8 (2.9 to 14.7)	1.37 (1.05 to 1.79)	6.5 (0.07 to 12.2)
Arts fac. (*n* = 295)	114 (38.6)	1.74 (1.38 to 2.21)	12.1 (6.5 to 17.7)	1.68 (1.31 to 2.14)	11.0 (5.3 to 16.6)
**Anxiety symptoms**					
Student pop. (*n* = 48,552)	12,145 (25.0)	1 (Reference)		1 (Reference)	
All music and arts (*n* = 877)	303 (34.6)	1.58 (1.37 to 1.82)	9.5 (6.4 to 12.7)	1.60 (1.38 to 1.85)	9.6 (6.3 to 12.8)
Music (*n* = 327)	93 (28.4)	1.19 (0.94 to 1.52)	3.4 (−1.5 to 8.4)	1.34 (1.04 to 1.71)	5.7 (0.5 to 10.9)
Perf. arts (*n* = 255)	99 (38.8)	1.90 (1.48 to 2.45)	13.8 (7.8 to 19.8)	1.78 (1.37 to 2.30)	11.9 (6.0 to 17.9)
Arts fac. (*n* = 295)	111 (37.6)	1.81 (1.43 to 2.29)	12.6 (7.1 to 18.2)	1.75 (1.37 to 2.24)	11.6 (6.0 to 17.3)
**Depressive symptoms**					
Student pop. (*n* = 48,568)	16,102 (33.2)	1 (Reference)		1 (Reference)	
All music and arts (*n* = 876)	362 (41.3)	1.42 (1.24 to 1.63)	8.2 (4.9 to 11.5)	1.41 (1.22 to 1.62)	7.9 (4.5 to 11.2)
Music (*n* = 326)	121 (37.2)	1.19 (0.95 to 1.49)	4.0 (−0.1 to 9.2)	1.28 (1.02 to 1.62)	5.7 (0.2 to 11.2)
Perf. arts (*n* = 255)	110 (43.1)	1.53 (1.19 to 1.96)	10.0 (3.9 to 16.1)	1.39 (1.08 to 1.79)	7.6 (1.5 to 13.7)
Arts fac. (*n* = 295)	131 (44.4)	1.61 (1.28 to 2.03)	11.3 (5.6 to 16.9)	1.57 (1.23 to 1.99)	10.5 (4.7 to 16.3)

*Estimated odds ratio (OR) and prevalence differences (PD) with 95% CI*.

a *Adjusted for Age, Sex, and Semesters of study*.

Music and arts students also reported higher prevalence of mental health disorders [PD 8.1 (CI 5.3–11.0)], which also was prevalent in anxiety disorders [PD 6.0 (CI 3.6–8.4)] and depressive disorders [PD 4.3 (CI 1.9–6.7)] compared to the general student population (Table 3). There were no substantial differences between students from music only faculties and the general student population with regards to depressive disorder.

**Table 3:** Logistic regression analysis of prevalence of symptoms of self-reported overall mental, anxiety, and depressive disorders in Norwegian music and arts students compared to the general student population.

		**Crude**		**Adjusted^a^**	
	**Prev. (%)**	**OR (CI)**	**PD (CI)**	**OR (CI)**	**PD (CI)**
**Mental disorders**					
Student pop. (*n* = 48,729)	7,519 (15.4)	1 (Reference)		1 (Reference)	
All music and arts (*n* = 880)	206 (23.4)	1.68 (1.43 to 1.96)	8.0 (5.2 to 10.8)	1.71 (1.46 to 2.01)	8.1 (5.3 to 11.0)
Music (*n* = 327)	56 (17.3)	1.13 (0.85 to 1.51)	1.7 (−2.4 to 5.8)	1.30 (0.97 to 1.73)	3.6 (−0.1 to 8.0)
Perf. arts (*n* = 256)	62 (24.2)	1.75 (1.31 to 2.33)	8.8 (3.5 to 14.1)	1.81 (1.35 to 2.41)	9.2 (3.9 to 14.4)
Arts fac. (*n* = 297)	88 (29.6)	2.31 (1.80 to 2.96)	14.2 (9.0 to 19.4)	2.10 (1.62 to 2.72)	12.0 (6.9 to 17.1)
**Anxiety disorders**					
Student pop. (*n* = 48,729)	4,868 (10.0)	1 (Reference)		1 (Reference)	
All music and arts (*n* = 880)	140 (15.9)	1.70 (1.42 to 2.05)	5.9 (3.5 to 8.4)	1.75 (1.45 to 2.10)	6.0 (3.6 to 8.4)
Music (*n* = 327)	39 (11.9)	1.22 (0.87 to 1.71)	1.9 (−1.6 to 5.5)	1.38 (0.99 to 1.94)	3.2 (−0.6 to 7.0)
Perf. arts (*n* =255)	49 (19.4)	2.13 (1.56 to 2.92)	9.2 (4.3 to 14.0)	2.14 (1.56 to 2.94)	8.9 (4.1 to 13.6)
Arts fac. (*n* = 295)	52 (17.5)	1.91 (1.42 to 2.58)	7.5 (3.2 to 11.9)	1.79 (1.32 to 2.44)	6.4 (2.2 to 10.5)
**Depressive disorder**					
Student pop. (*n* = 48,729)	5,375 (11.0)	1 (Reference)		1 (Reference)	
All music and arts (*n* = 876)	135 (15.3)	1.46 (1.21 to 1.76)	4.3 (1.9 to 6.7)	1.48 (1.22 to 1.79)	4.3 (1.9 to 6.7)
Music (*n* = 327)	31 (9.5)	0.84 (0.58 to 1.22)	−1.6 (−4.7 to 1.6)	0.95 (0.66 to 1.38)	−0.4 (−3.9 to 3.0)
Perf. arts (*n* = 256)	42 (16.4)	1.58 (1.13 to 2.21)	5.4 (0.8 to 9.9)	1.63 (1.17 to 2.28)	5.6 (1.1 to 10.2)
Arts fac. (*n* = 297)	62 (20.9)	2.12 (1.61 to 2.82)	9.9 (5.2 to 14.5)	1.92 (1.44 to 2.58)	8.0 (3.6 to 12.4)

*Estimated odds ratio (OR) and prevalence differences (PD) with 95% CI*.

a *Adjusted for Age, Sex, and Semesters of study*.

In term of psychotherapy use (Table 4), defined by current contact with a psychologist and/or psychiatrist, there was a statistically significant difference between music and arts students and the general student population [PD 7.4 (CI 4.9–9.9)], which also was seen across all faculty affiliations.

**Table 4:** Logistic regression analysis of use of psychotherapy (psychologist and/or psychiatrist) in Norwegian music and arts students compared to the general student population.

		**Crude**	**Adjusted^a^**		
	**Prev. (%)**	**OR (CI)**	**PD (CI)**	**OR (CI)**	**PD (CI)**
**Psychotherapy**					
Student pop. (*n* = 48,729)	5,024 (10.3)	1 (Reference)		1 (Reference)	
All music and arts (*n* = 880)	156 (17.7)	1.87 (1.57 to 2.33)	7.4 (4.9 to 10.0)	1.91 (1.60 to 2.29)	7.4 (4.9 to 9.9)
Music (*n* = 327)	51 (15.6)	1.61 (1.19 to 2.17)	5.3 (1.4 to 9.3)	1.87 (1.38 to 2.53)	7.1 (2.9 to 11.4)
Perf. arts (*n* = 256)	43 (16.8)	1.76 (1.26 to 2.44)	6.5 (1.9 to 11.1)	1.83 (1.31 to 2.56)	6.9 (2.2 to 11.5)
Arts fac. (*n* = 297)	62 (20.9)	2.30 (1.73 to 3.04)	10.6 (6.0 to 15.3)	2.02 (1.50 to 2.71)	8.2 (3.9 to 12.6)

*Estimated odds ratio (OR) and prevalence differences (PD) with 95% CI*.

a *Adjusted for Age, Sex, and Semesters of study*.

## Discussion

In this national health survey of all Norwegian full-time students pursuing higher education, anxiety, and depression symptoms were highly prevalent among music and art students compared to the general student population. The same differences were observed in terms of self-reported mental disorders and use of psychotherapy. Even though the data show an elevated degree of anxiety and depression symptoms and psychotherapy use among students within music and arts, a small tendency of a gradient toward less difference were observed for those who were affiliated to music only faculties. Nevertheless, the overall findings should be interpreted with caution due to the limitations listed below.

### Strengths and Limitations

This is, to our best knowledge, the first large-scale investigation of its kind. A large sample size made it possible for us to look at differences in prevalence rates using a validated instrument.

A major limitation in this study is the cross-sectional design. The lack of repeated measures makes it difficult to establish conclusions regarding causal relationships and underlying mechanisms explaining the observed associations. Another limitation is the low response rate (31%), but at least this was relatively consistent across all universities, making it relevant to review the relative differences between groups of students.

Further, to identify music and performing art students, we had to rely upon using institutions and/or faculties which were dedicated to music and arts. Due to this, we were not able to include faculties and institutions that had placed their music and arts students within broader faculties such as the humanistic faculty. In other words, a proportion of the music and arts students in Norway has not been included, and rather been used as controls together with students of the general student population in this study. By dividing our music and art sample into music faculties, performance faculties and music and art faculties, based on their name, we have found that there seems to be some discrepancies, and that our sample is somewhat heterogenic. Hence, the results should be read with caution. Self-report bias is also a well-known limitation with regard to use of questionnaires (Razavi, 2001), which also is the case in our study. Especially this is important to take into account when interpreting our results on self-reported mental health disorders and use of psychotherapy.

## General Discussion

First and foremost, our results are in line with previous large-scale studies with regard to prevalence of anxiety and depression symptoms (Vaag et al., 2016a), and use of psychotherapy among professional musicians (Vaag et al., 2016b) compared to the general population. The discrepancies between music and arts students and the general student population are a bit lower than found when comparing professional musicians to the general workforce, this may be due to the healthy worker effect? that often operate over time in the workforce, which cannot be expected in a student sample.

As mentioned in the introduction, a recent study by Kegelaers et al. (2020) indicated that music students experienced high proportions of anxiety and depression symptoms, and significantly more symptoms than professional musicians. Based on our results, we see the same pattern of discrepancies between music and arts students and the general student population, and there seems to be a similar pattern of discrepancies in mental health symptoms and healthcare use as in studies comparing musicians to the general workforce (Vaag et al., 2016a,b). In contrast, although with the lack of control groups, Wristen (2013) did not find higher levels of anxiety and depression symptoms in music students than the general student population. Even though the current study does find statistically significant group differences, we acknowledge the fact that our employed categorization is based on a combination of music and arts students, and a more fine-meshed grouping of students might have yielded more specific results. Especially due to the fact that if one looks more closely into the results, there seems to be a tendency that students from “music only” faculties report less symptom loads than the students from combined faculties. And even though there are no differences with regard to use of psychotherapy, there are undoubtedly variations within the music and arts students included in our study, particularly in self-reported depressive disorder, where music only students did not differ from the general student population (even though differences were found in self-reported depressive symptoms using the scale measurement).

Students and professionals from the music and art education and music profession seem to describe high symptom loads of anxiety and depression, where mental illness and use of psychotherapy seems to follow the same pattern. A major question is whether this is due to the unique contextual properties that follows the education and profession, such as a high degree of competition, performance pressure (Chong et al., 1989; Van Kemenade et al., 1995; Langendörfer et al., 2006; Dobson, 2010; Barbar et al., 2014; Kenny et al., 2014) and demands (Holst et al., 2012; Vaag et al., 2013; Aalberg et al., 2019; Detari et al., 2020). If so, these findings support the notion that the industry is in need for transformation (Gross and Musgrave, 2020). Or, on the other hand, the discrepancies could rather be attributed to the individual characteristics of people attracted to the profession, and the proposed association between creativity and mental illness indicated in large-scale epidemiological studies (Bellis et al., 2007; Kyaga et al., 2013), and an indication of increased prevalence of the personality trait neuroticism among musicians (Cooper and Wills, 1989; Kemp, 1996; Gillespie and Myors, 2000; Vaag et al., 2018).

From a research perspective, there is a need for long-term investigations of both career and health patterns. From a practice perspective, there is a growing amount of research indicating that it is important to plan for health promotive and preventative measures within both the education and professional industry. Research on the possible effects of different interventions, structural and organizational changes is warranted. Our study mainly focused on musicians, yet these findings also underline a need to further investigate mental health in the same manner within other areas of the performing arts, using more fine-meshed epidemiological cohorts.

## Conclusion

Our results show considerably higher prevalence rates of anxiety and depression symptoms among Norwegian music and art students, compared to the general student population. We find the same differences for self-reported anxiety and depression disorders, and use of psychotherapy. Our findings are consistent with previous results reporting similar discrepancies between musicians and the general workforce. The results encourage further work with preventative measures within the educational system and industry, more fine-meshed studies into the health challenges of students and professionals within the arts, as well as further research using prospective mental health data and its correlates among aspiring musicians and artists.

## Data Availability Statement

Norwegian data protection regulations and GDPR impose restrictions on sharing of individual participant data. However, researchers may gain access to survey participant data by contacting the publication committee (borge.sivertsen@fhi.no). Approval from the Norwegian Regional Committee for Medical and Health Research Ethics (https://helseforskning.etikkom.no) is a pre-requirement for access to the data. The dataset is administrated by the NIPH, and guidelines for access to data are found at https://www.fhi.no/en/more/access-to-data.

## Ethics Statement

The studies involving human participants were reviewed and approved by Regional Committee for Medical and Health Research Ethics in Western Norway. The patients/participants provided their written informed consent to participate in this study.

## Author Contributions

JV planned the study, conducted the analysis, and wrote the manuscript. OB contributed to planning the study, gave insight into interpretation of analysis, and commented and reviewed the manuscript. BS lead the data collection, contributed to planning the study, gave insight into interpretation of analysis, and commented and reviewed the manuscript. All authors contributed to the article and approved the submitted version.

## Conflict of Interest

The authors declare that the research was conducted in the absence of any commercial or financial relationships that could be construed as a potential conflict of interest.
